# Brain diffusion tensor imaging reveals altered connections and networks in epilepsy patients

**DOI:** 10.3389/fnhum.2023.1142408

**Published:** 2023-03-22

**Authors:** Meixia Wang, Xiaoyu Cheng, Qianru Shi, Bo Xu, Xiaoxia Hou, Huimin Zhao, Qian Gui, Guanhui Wu, Xiaofeng Dong, Qinrong Xu, Mingqiang Shen, Qingzhang Cheng, Shouru Xue, Hongxuan Feng, Zhiliang Ding

**Affiliations:** ^1^Department of Neurology, Suzhou Hospital Affiliated to Nanjing Medical University, Suzhou Municipal Hospital, Suzhou, China; ^2^Department of Neurology and Clinical Research Center of Neurological Disease, The Second Affiliated Hospital of Soochow University, Suzhou, China; ^3^Department of Neurology, The First Affiliated Hospital of Soochow University, Suzhou, China

**Keywords:** DTI, brain network, epilepsy, graph theory, white matter fiber connectivity

## Abstract

**Introduction:**

Accumulating evidence shows that epilepsy is a disease caused by brain network dysfunction. This study explored changes in brain network structure in epilepsy patients based on graph analysis of diffusion tensor imaging data.

**Methods:**

The brain structure networks of 42 healthy control individuals and 26 epilepsy patients were constructed. Using graph theory analysis, global and local network topology parameters of the brain structure network were calculated, and changes in global and local characteristics of the brain network in epilepsy patients were quantitatively analyzed.

**Results:**

Compared with the healthy control group, the epilepsy patient group showed lower global efficiency, local efficiency, clustering coefficient, and a longer shortest path length. Both healthy control individuals and epilepsy patients showed small-world attributes, with no significant difference between groups. The epilepsy patient group showed lower nodal local efficiency and nodal clustering coefficient in the right olfactory cortex and right rectus and lower nodal degree centrality in the right olfactory cortex and the left paracentral lobular compared with the healthy control group. In addition, the epilepsy patient group showed a smaller fiber number of edges in specific regions of the frontal lobe, temporal lobe, and default mode network, indicating reduced connection strength.

**Discussion:**

Epilepsy patients exhibited lower global and local brain network properties as well as reduced white matter fiber connectivity in key brain regions. These findings further support the idea that epilepsy is a brain network disorder.

## 1. Introduction

Epilepsy is a brain function disorder caused by excessive discharge of neurons. Epilepsy patients often show neurological problems such as poor language ability, impaired memory, attention deficits, mental decline, and impaired executive function. Traditionally, the onset of epilepsy is believed to be due to electrical dysfunction. However, as brain regions work in coordination as a whole network, accumulating evidence indicates that epilepsy and other diseases of the central nervous system involve brain network dysfunction (Royer et al., 2022). In particular, numerous animal models and human experiments show that the cortical-subcortical network plays a crucial role in the propagation and behavioral manifestation of epilepsy (Bernhardt et al., 2015). How focal seizures spread to distant brain regions also suggests a link between brain network dysfunction and structural abnormalities (Chen et al., 2015). However, a complete understanding of the characteristics of the brain network in epilepsy requires further exploration.

The structural basis of the brain network is the connection of white matter fiber tracts across multiple functional brain regions (Posner et al., 2006). Diffusion tensor imaging (DTI) is a non-invasive magnetic resonance imaging (MRI) technique that provides information on the microstructural features of deep tissue in the living human brain. During the diffusion process, water molecules in myelinated nerve fibers show obvious directionality due to restriction of the myelin sheath (Konomi et al., 2012). Thus, DTI images enable observation of the fine structure of white matter that cannot be seen by MRI, allowing earlier and more sensitive detection of lesions, meeting the needs of auxiliary guidance for epilepsy surgery, and supporting investigations of the structural brain network and its role in cognitive dysfunction in epilepsy (Yao et al., 2018).

At present, the evaluation of complex structural brain networks is mainly based on graph theory, in which is the brain network transformed into nodes (i.e., cerebral gray matter areas) and links (i.e., white matter connections between the brain gray matter areas), and information processing and transmission properties of the network are measured through topological parameters (Mears and Pollard, 2016). Topological features of structural connections can intuitively describe complex brain networks and provide a new way to understand the healthy brain as well as brains with neuropathological changes (Ji et al., 2019). Multiple studies show that changes in brain DTI parameters are associated with the degree of clinical deficits in epilepsy patients (A Yassine et al., 2018). In addition, other studies conclude that changes in network topological parameters imply various types of abnormalities in structural connectivity in epilepsy patients (Liu et al., 2014). These findings confirm the close association between epilepsy patient dysfunction and structural changes in the brain, which are likely to affect information transmission pathways in the brain network.

Hub nodes, which are highly connected regions in the brain network that can be identified by graph theoretical approaches, play a central role in integrating and rapidly transmitting information with minimal energy cost (Bullmore and Sporns, 2009). These hub nodes increase the brain’s integrative capacity through their high connectivity, short neuronal pathways, and high centrality (Sporns, 2014). However, few studies have investigated hub node reorganization within subcortical structures in epilepsy (Mears and Pollard, 2016). Thus, network topology parameters require further study to more comprehensively characterize the brain network of epilepsy patients.

In this study, we constructed a structural brain network based on DTI and the fiber tract tracking method, calculated network topology parameters of the whole-brain network and local brain areas, quantitatively analyzed global and local network characteristics, and evaluated changes in the microstructure of the brain in epilepsy patients and healthy control individuals. The purpose of this project is to explore the microstructure changes of epilepsy patients, so as to further explore the potential pathophysiological changes in epilepsy and provide basis for early diagnosis.

## 2. Materials and methods

### 2.1. Participants

A total of 26 epilepsy patients and 42 healthy control individuals were recruited randomly from the Epilepsy Clinic of Suzhou Municipal Hospital from June 2018 to April 2022. Epilepsy patients with different seizure types are included, such as simple partial seizures, complex partial seizures and generalized seizures. All patients underwent standard clinical assessment, including detailed seizure history, neurological examination, neuropsychological assessment, standard and video-electroencephalography (EEG) evaluation, and brain MRI. Patients were included if they: (1) met the 2015 International League Against Epilepsy diagnostic criteria for epilepsy, (2) had an intelligence level sufficient to understand and complete the various scales employed in the study, (3) were 22–65 years old, and (4) agreed to participate in the study. Patients were excluded if they: (1) had severe blood system disease; heart, liver, or kidney dysfunction; tumors; thyroid dysfunction; or organ transplantation or (2) had obvious developmental delay, inability to cooperate, mental illness, or traumatic brain injury. The healthy control group consisted of 42 healthy volunteers matched on age and mean number of educational years. No healthy control individual had a history of significant medical, neurological, or psychiatric disorders. To avoid any confounding effects on cognition, all participants were assessed using the Mini-Mental State Examination (MMSE), Hamilton Anxiety Rating Scale (HAM-A), and Hamilton Depression Scale (HAMD-17).

The efficacy analysis was performed by using R language pwr package *t*-test. The sample size of the control group is 42, and the sample size of the epilepsy group is 26, α = 0.05, according to the pre-test study, it was found that the two groups of global efficiency *d* = 0.75, and power = 84.17% was calculated.

This study was approved by the ethics committee of Suzhou Municipal Hospital, an affiliated hospital of Nanjing Medical University (Ethical review No. K–2022–050–K01; Date of ethical review, March 10, 2022). All participants were right-handed and provided informed consent prior to the study.

### 2.2. Image acquisition

Diffusion tensor imaging data were obtained on a 3.0-Tesla MRI scanner (GE DISCOVERY MR750). Scanning was performed with participants in a supine position with their head fixed while awake with their eyes closed. A high-resolution structural image was acquired for each participant with a whole-brain T1-weighted scan (TR/TE = 2,300/2.98 ms, flip angle = 9°, slice thickness = 1.10 mm (no gap), field of view = 248 × 248 mm, matrix = 248 × 256) for spatial brain normalization. The structural map was screened by imaging staff with at least 10 years of experience, and participants with obvious intracranial organic lesions were excluded from further study.

All participants also underwent DTI to obtain data suitable for graph theoretical analysis. DTI data were acquired using spin-echo single-shot echo-planar pulse sequences with the following parameters: TR/TE = 5,400/93 ms, flip angle = 90°, field of view = 220 × 220 mm, slice thickness = 4 mm (no gap), matrix = 122 × 122, and *b* = 1,000 s/mm^2^. Diffusion gradients were applied in 30 different non-linear directions. Raw DTI image files were transferred from the scanner to a computer. All image acquisitions were performed using the same scanner by trained technicians. All the subjects were conscious during the examination.

### 2.3. Data pre-processing

Diffusion tensor imaging data were preprocessed using the Pipeline for Analyzing braiN Diffusion imAges (PANDA) toolkit (Cui et al., 2013) in MATLAB including the following steps: converting DICOM image files into NIfTI format, estimating the whole-brain mask, and performing eddy current and head motion corrections. Calculation of eddy current and head motion corrections was performed by applying an affine alignment of the diffusion-weighted images to b0 images using tFMRIB (FMRIB’s Software Library, FSL Diffusion Toolbox, version 5.0)^1^.

### 2.4. Construction of brain networks

Nodes and edges are the two basic elements of a network. In this study, each participant’s white matter structural network was constructed by defining the network’s nodes and edges. When the PANDA toolkit performs deterministic white matter fiber tracing of diffusion tensor data, the brain is divided into 90 regions that serve as nodes in the brain network, and the white matter fiber tracts connecting these nodes are considered edges. To define network nodes, individual T1-weighted images were non-linearly registered to the standard space using the PANDA toolkit to obtain the inverse transform T-1, which was then applied to the selected template to obtain 90 brain regions based on the Automated Anatomic Labeling-90 (AAL-90) Atlas (Tzourio-Mazoyer et al., 2002). The fiber assignment by continuous tracking (FACT) algorithm was used for deterministic fiber tracking in PANDA. The tracking of each fiber stopped when the deflection angle exceeded 45° or the fractional anisotropy value was <0.2. To avoid false-positive results, we kept only those fiber connections that existed in >80% of participants and had a fiber number of ≥3 (Shu et al., 2011).

### 2.5. Graph theory analysis

Calculation and statistical analysis of whole-brain and local-regional graph theoretic indicators were performed using Gretna software on the MATLAB platform.^2^ After setting the fiber number threshold to 3 (if the fiber network was ≥3, the edge connecting weight was set to 1; otherwise, it was set to 0), we built a binary network and then calculated various graph theory indicators.

#### 2.5.1. Global network properties

Global network properties included the following measures:

Global efficiency (E_global_) is the reciprocal average of the shortest path length of all paths between each pair of nodes in the entire network. This measure indicates the information transmission ability between nodes in the network. The shorter the shortest path length, the higher the global efficiency of the network, and the faster the speed of information transfer between nodes. The formula is as follows:


$$E_{\text{global}}=\frac{1}{N\,\left(N-1\right)}\,\sum_{i,j,i\neq j}^{n}\frac{1}{d_{i\,j}}$$

where N represents the number of nodes, and d_*ij*_ represents the distance between node i and node j.

Local efficiency (E_local_) is the global efficiency of each sub-network G_*i*_. This measure indicates the information transmission capability of nodes in the network. The formula is as follows:


$$E_{\text{local}}\,\left(i\right)=E_{\text{global}}\,\left(G_{i}\right)$$

Clustering coefficient (C_*i*_) is the ratio of the number of edges between other nodes directly connected to a node in the network to the maximum possible number of edges among these other nodes. This measure indicates the degree of groupization of the network. The formula is as follows:


$$C_{i}=\frac{E_{i}}{\frac{1}{2}\,k\,i\,\left(k\,i-1\right)}$$

where E_*i*_ represents the number of edges between other nodes directly connected to node i, and k_*i*_ represents the degree of connectivity of node i. k_*i*_ (k_*i*_-1)/2 is the maximum number of connected edges of other nodes. It can be seen from the formula that C_*i*_ measures the grouping degree of the node.

Shortest path length (L_*p*_) is the average of the shortest paths from a node to all other nodes in the network (Shah et al., 2017). The formula is as follows:


$$L_{i}=\frac{1}{N-1}\,\sum_{i\neq j\in G}L_{i,j}$$

where L_*i,j*_ represents the shortest path between node i and node j. This measure indicates how tightly the network is connected.

A small-world network is a network model with a high clustering coefficient, similar to a regular network, as well as a short shortest path length, similar to a random network. The small-world property is measured by σ = λ/γ, where σ is defined by the clustering coefficient (C_*p*_) and the shortest path length (L_*p*_). A network is considered to have small-world properties when the network clustering coefficient C_real_ is greater than the random network clustering coefficient C_random_ (γ = C_real_/C_random_, γ > 1), when path length L_real_ is equal to the random network path length L_random_ (λ = L_real_/L_random_, λ≈1), or when σ = λ/γ > 1. C_random_ is the average clustering coefficient of 500 random networks, and L_random_ is the average shortest path length of 500 random networks.

#### 2.5.2. Node properties

Node properties included the following measures:

The global efficiency of node i (E_glob_i_) measures the ability of node i to transmit information across the network. The formula is as follows:


Eglob_i=(1/N-1)*Σ(1/L)i,j

where L_*i,j*_ represents the shortest node i path between and node j. The larger E_glob_i_, the faster the information transmission between node i and other nodes.

The local efficiency of node i (E_loc_i_) measures the compactness of a small network composed of nodes adjacent to node i. The formula is as follows:


$$E_{\mathrm{loc}\,\_\,i}=\frac{1}{N_{G\,i}\,\left(N_{G\,i}-1\right)}\,\sum_{j.k\in G\,i}^{n}\frac{1}{L_{j.k}}$$

where G_*i*_ represents the sub-network composed of other nodes directly connected to node i, N_*Gi*_ is the total number of nodes in the sub-network, and L_*j,k*_ represents the shortest path between node j and node k.

Node degree centrality (K_*i*_) is the number of edges that node i shares with other nodes in the network. The formula is as follows:


$$K_{i}=1/n\,\Sigma \,k_{i}$$

This measure indicates the importance of a single node in the network.

Betweenness centrality [N_*bc*_ (i)] is the number of paths passing through a node that are part of the shortest paths between all other pairs of nodes in the entire network. The formula is as follows:


$$N_{b\,c}\,\left(i\right)=\sum_{j\neq i\neq k\in G}^{n}\frac{\delta_{j\,k}\,\left(i\right)}{\delta_{j\,k}}$$

where δ_*jk*_ represents the number of shortest paths between any two nodes except node i in the connection network and δ_*jk*_ (i) is the number of paths passing through node i that are part of the shortest path between any two other nodes. This measures the importance of node i in the network.

### 2.6. Edge analysis

To further characterize changes in white matter structural connection strengths in specific brain regions, the network-based statistic approach was used as described by Zalesky et al. (2010).

### 2.7. AutoPTX analysis

First, FSL software was used to preprocess the data of each subject, including head-motion eddy current correction, gradient direction correction, and obtain the brain range mask. Then the autoPtx tool was used to calculate index the dispersion, get the fractional anisotropy (FA) index, estimate the BedPostX direction distribution, and register the DTI space-standard space. Finally, fiber bundles were obtained by probabilistic fiber tracking. The average FA index value within the range of fiber bundle mask of each subject was extracted, and the two-sample *T*-test (using matlab code for statistics) was carried out for the two groups to obtain the *p*-value and corresponding *T*-value. The results were corrected by Bonferoni (*p* < 0.05).

### 2.8. Statistical analysis

Statistical analysis was performed using IBM SPSS statistics (version 22). Clinical data with a normal distribution are expressed as mean ± standard deviation and were analyzed using two-sample *t*-tests to test for differences between epilepsy patient and healthy control groups. Frequency data are expressed as rate and percentage and were analyzed using chi-square tests to test for differences between groups. A *p*-value < 0.05 was considered a statistically significant difference. Graph measurements were analyzed with ANCOVA to detect differences between epilepsy patient and healthy control groups over a wide range of thresholds. The false discovery rate (FDR) procedure was employed to correct for multiple comparisons in global and local network analyses.

## 3. Results

### 3.1. Demographic characteristics

There were no significant differences between epilepsy patient and healthy control groups in age, sex, educational level, or cognitive performance (Table 1).

**TABLE 1:** Demographic and clinical characteristics of participants.

Variables	Healthy controls (*n* = 42)	Epilepsy patients (*n* = 26)	*t* or X^2^ value	*P*-value
Age (years)	27.69 ± 6.73	27.77 ± 5.31	−0.051	0.96
Sex (number of males/females)	19/23	16/10	−1.304	0.197
Education level (years)	11.85 ± 3.17	11.61 ± 2.58	0.327	0.745
Duration of epilepsy (years)	–	6 ± 5.19	–	–
Seizure type	–	–	–	–
Focal seizures	–	17 (65.38)	–	–
Generalized seizures	–	11 (34.62)	–	–
Seizure frequency (per month)	–	–	–	–
≤1 time	–	16 (61.54)	–	–
≥1 time	–	10 (38.46)	–	–
Antiepileptic drugs	–	–	–	–
Monotherapy	–	19 (73.08)	–	–
Polytherapy	–	7 (26.92)	–	–
HAM-A	6.38 ± 1.92	6.31 ± 1.67	0.16	0.873
HAMD-17	6.26 ± 1.87	6.20 ± 2.76	−0.104	0.918
MMSE	28.12 ± 1.37	27.92 ± 1.49	0.555	0.581

Data are expressed as mean ± standard deviation or number (percentage). MMSE, Mini-Mental State Examination; HAM-A, Hamilton Anxiety Rating Scale; HAMD-17, Hamilton Depression Scale-17.

### 3.2. Global network properties

**FIGURE 1:**
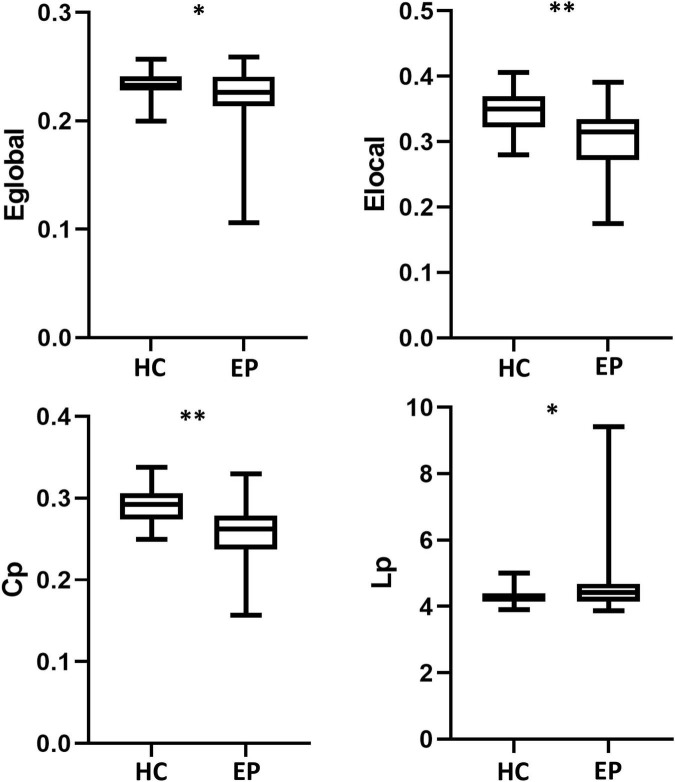
Global network measures. Global efficiency (E the epile_global_), local efficiency (E_local_), and clustering coefficient (C_p_) were significantly lower in epilepsy patient (EP) group than in the healthy control (HC) group. Shortest path length (L_p_) was longer in the EP group than in the HC group. *p < 0.05, ^**^p < 0.01, false discovery rate-corrected.

Compared with the healthy control group, the epilepsy patient group had lower global and local efficiency, a smaller clustering coefficient, and a longer shortest path length (*p* < 0.05, FDR-corrected) (Figure 1). Small-world attributes were significantly greater than one in both groups, with no significant differences in these attributes between groups (*p* > 0.05, FDR-corrected) (Table 2).

**TABLE 2:** Global network topological properties and small-world properties.

	Healthy controls (*n* = 42)	Epilepsy patients (*n* = 26)	*t* or X^2^ value	*P*-value
Global efficiency (E_global_)	0.234 ± 0.012	0.221 ± 0.031	2.344	0.022
Local efficiency (E_local_)	0.345 ± 0.029	0.305 ± 0.050	4.25	< *0*.01
Clustering coefficient (C_p_)	0.291 ± 0.022	0.257 ± 0.037	4.681	< *0*.01
Shortest path length (L_p_)	4.287 ± 0.219	4.651 ± 1.057	−2.168	0.034
Sigma	5.974 ± 0.571	5.857 ± 0.621	0.789	0.433
Gamma	7.836 ± 0.725	7.668 ± 0.935	0.833	0.408
Lambda	1.313 ± 0.033	1.308 ± 0.058	0.452	0.653

All data are expressed as mean ± standard deviation. Small-world properties were measured by σ = λ/γ. False discovery rate correction was applied to correct for multiple comparisons.

### 3.3. Node properties

**FIGURE 2:**
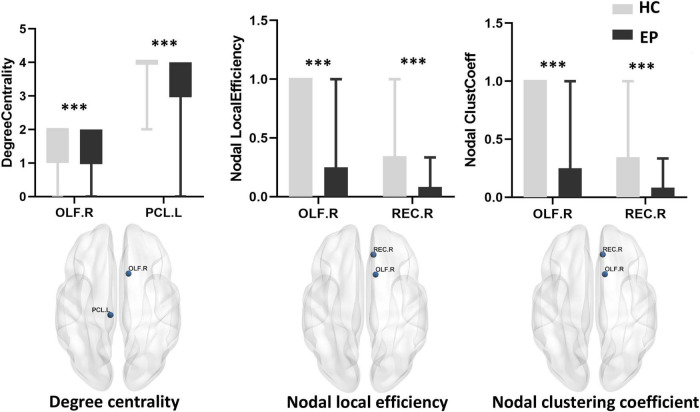
Local network measures of subcortical structures between healthy control (HC) and epilepsy patient (EP) groups, including nodal degree centrality, nodal local efficiency, and nodal clustering coefficient. The corresponding MRI graphic construction is below the box plot graphs. No significant main group effect was found for betweenness centrality. ^***^p < 0.001, false discovery rate-corrected. OLF.R, right olfactory cortex; PCL.L, left paracentral lobule; REC.R, right rectus.

Compared with the healthy control group, the epilepsy patient group showed lower nodal degree centrality in the right olfactory cortex (OLF.R) and left paracentral lobule (PCL.L) as well as lower nodal local efficiency and smaller nodal clustering coefficients in the right olfactory cortex (OLF.R) and right rectus (REC.R) (*p* < 0.001, FDR-corrected) (Figure 2 and Table 3). No significant difference between groups was found for betweenness centrality (*p* > 0.05, FDR-corrected).

**TABLE 3:** Local network properties.

	Degree centrality		Nodal local efficiency		Nodal clustering coefficient	
	**OLF.R**	**PCL.L**	**OLF.R**	**REC.R**	**OLF.R**	**REC.R**
*t*-value	3.694	4.142	4.059	3.677	4.059	3.677
*P*-value	<*0*.001	<*0*.001	<*0*.001	<*0*.001	<*0*.001	<*0*.001

OLF.R, right olfactory cortex; PCL.L, left paracentral lobule; REC.R, right rectus. *t*-values were obtained from two-sample *t*-tests. False discovery rate correction was applied to correct for multiple comparisons.

### 3.4. Edge analysis

**FIGURE 3:**
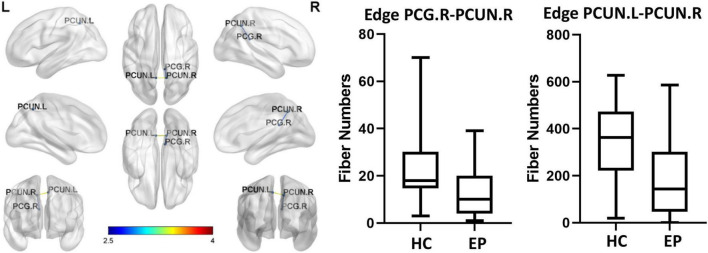
Brain network consisting of two edges connecting three different regions. The two pairs of regions exhibited significantly less connectivity in the epilepsy patient (EP) group than in the healthy control (HC) group (corrected *p* = 0.017, false discovery rate-corrected). PCUN.L, left precuneus; PCUN.R, right precuneus; PCG.R, right post cingulum.

Edge analysis was performed based on fiber number using two-sample *t*-tests with the network-based statistic correction method (edge *p*-value set to <0.01, component *p*-value set to <0.05, 5,000 permutations). This approach identified two subnetworks of reduced connectivity in epilepsy patients (*p* < 0.05, corrected for multiple comparisons). The circuit consisted of two edges connecting three regions. These edges connected the left precuneus (PCUN.L) to the right precuneus (PCUN.R) and the right precuneus (PCUN.R) to the right post cingulum (PCG.R). Compared with the healthy control group, the epilepsy patient group had a smaller fiber number (*p* = 0.017, FDR-corrected, Figure 3).

### 3.5. AutoPTX analysis

**FIGURE 4:**
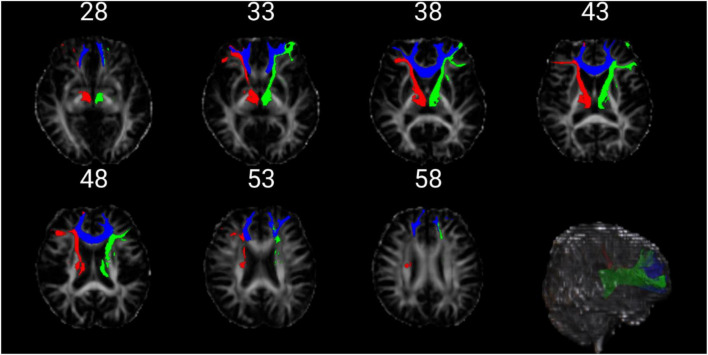
The figure above shows the position of the three fibers. The red is atr_l. The green is atr_r. The blue is fmi.

The FA index (Bonferoni corrected) showed significant differences in three fiber bundles, which were the left Anterior thalamic radiation (atr_l), the right Anterior thalamic radiation (atr_r), and the Forceps minor (fmi). The FA value of the three fiber bundles in the epilepsy group was significantly lower than that in the healthy control group (*p* < 0.001) (Figure 4 and Table 4).

**TABLE 4:** AutoPTX analysis.

Fiber bundle	atr_l	atr_r	fmi
*t*-value	3.689	4.021	3.76
*P*-value	<0.001	<*0*.001	<0.001

Comparison of FA values (art_l, art_r, and fmi) bundles between EP group and HC group. atr_l, the left anterior thalamic radiation; atr_r, the right anterior thalamic radiation; fmi, the forceps minor. The two groups did two-sample *T*-test (matlab code was used), and the *p*-value and corresponding *t*-value were obtained. The results were corrected by Bonferoni (*p* < 0.001). False discovery rate correction was applied to correct for multiple comparisons.

## 4. Discussion

Epilepsy is a brain function disorder caused by the excessive discharge of neurons. Recently, a study has shown that long-term and recurrent seizures can lead to changes in the topological properties of the whole or local brain regions (Park et al., 2019). Our study explored changes in DTI network characteristics of the entire brain and specific brain regions in epilepsy patients. We found that brain networks in epilepsy patients show changes in network structure and information transfer capacity.

In the comparison of global properties, we found that the brains of epilepsy patients showed significantly smaller clustering coefficients, longer shortest path lengths, and lower local and global efficiencies. These changes are reflective of an abnormal topology network and are consistent with those of Jiang et al. (2017). Global efficiency represents the ability of information conversion and transmission across brain nodes, with lower global efficiency suggesting a decline in node function throughout the brain. The increase in shortest path length indicates that information transferred from one node to another must pass through a larger number of links, suggesting a decreased tightness of network connections (Fang et al., 2022). As shorter path lengths ensure effective and convenient communication between brain regions (Shah et al., 2017), an increased shortest path length would inevitably lead to a decrease in global efficiency.

Small-world networks combine the topological advantages of regular and random networks, ensuring efficiency of information transfer at local and global levels (Hu et al., 2015). Our findings indicate that the brain network of epilepsy patients has small-world properties, consistent with the results of previous studies (Nemzer et al., 2020; Shigemoto et al., 2021). We also found no significant difference in small-world properties between epilepsy patients and healthy control individuals, thereby the small-world characteristics of the brain in epilepsy patients maintained, indicating that information transmitted between brain regions in epilepsy patients can occur through new optimal paths, reflecting the brain’s ability to separate and integrate information (Zhu et al., 2021).

Critical nodes play important roles in the transmission of information across the brain network by determining the integration efficiency of neural information in the network and the stability of network structure (Fransson and Thompson, 2020). In the present study, we found the same trend for local network features measured at key nodes as for the whole-brain network. This phenomenon implies that some selected key nodes have consistent transmission capabilities (Wu et al., 2013). The nodal local efficiency and nodal clustering coefficient in the epilepsy patient group were lower than those in the healthy control group, indicating reduced information transmission ability in the local brain areas. Also, nodal degree centrality in the epilepsy patient group was lower than that in the healthy control group, reflecting the functional decline of local brain regions in the whole network. In our study, nodes with these significant differences (e.g., olfactory cortex, paracentral lobule, rectus gyrus) were distributed in the frontal lobe, temporal lobe, and default mode network. The default mode network is involved in many functions such as emotion, cognition, and self-awareness maintenance (Svob et al., 2016), and frontal, temporal, and subcortical networks are critical for emotional regulation and cognitive function. Based on these findings, we speculate that the reduced efficiency of these brain regions in transmitting information is due to the destructive effects of the repeated epileptiform activities on the functional coordination of emotional/memory/cognitive-related networks (Tong et al., 2019), resulting in emotional, memory and cognitive dysfunction.

Anatomical connections between the olfactory cortex and other brain regions have been revealed by microscopic imaging and tracer studies, and macroscopic anatomical imaging demonstrates changes in the olfactory cortex in epilepsy (Sarnat and Flores-Sarnat, 2016; Menassa et al., 2017; Young et al., 2019), consistent with our findings. Moreover, expression of the epilepsy-related molecule DKK-1 was first induced in neurons of the olfactory cortex in an *in vivo* epilepsy model, which may explain the susceptibility of the olfactory cortex (Busceti et al., 2007). EEG analysis shows abnormal electrical activity originating from the right rectus in some epilepsy cases (Zhang et al., 2019). Thus, further studies of network connections in the right rectus region will help increase our understanding of the diffusion of electrical activity and clinical phenotypes in epilepsy. When the paracentral lobule is involved, the threshold of epilepsy in this area is low and can spread to a wider epileptogenic area (Hu et al., 2017). However, the underlying mechanism requires further exploration, and the analysis of network properties in the paracentral lobule may contribute to the unveiling of this phenomenon.

In recent years, we have recognized that epilepsy affects the white matter network of the brain, which is typically characterized by the loss of white matter microstructure and the interruption of network connection (Hatton et al., 2020). In this study, we found that the number of connections between the right PCG and bilateral PCUN was significantly smaller in the epilepsy patient group than in the healthy control group. Functionally, the PCUN is associated with many high-level cognitive functions such as episodic memory and self-related information processing, whereas the PCG is associated with visual information transfer. A decrease in fiber connections in these regions will directly lead to dysfunction of their conduction pathways (Xu et al., 2021), which will further lead to a decline in learning, memory, cognition, and other abilities. A study of functional MRI (fMRI) in epilepsy patients shows an abnormal functional connection between the PCG/PCUN and the whole brain (McGill et al., 2012), which is mutually supportive of our results. A study based on EEG-fMRI found that epileptiform discharges are accompanied by functional responses in the precuneus (Fahoum et al., 2012), suggesting that the precuneus plays a role in the propagation of epileptic discharges. The present study suggests that the structural integrity of white matter fibers in the precuneus may be disrupted during epileptiform discharges, which may be responsible for a decline in the function of fiber connections in key brain regions.

Consistent with some previous results, our results suggest that the FA value of epilepsy patients decreases (Gong et al., 2017). FA value is used to measure the ability of directional diffusion of water molecules in tissues. The damage of white matter fiber structure in epileptic patients reduces the anisotropy of water molecule dispersion, leading to the decrease of FA value (Xu et al., 2018). In our study, the white matter fibers involved in epileptic patients include the left and right anterior thalamic radiation and forceps minor. The anterior thalamic radiation and their interconnecting fibers, which are important components of an extended hippocampal circuit for episodic memory, pass through the temporal lobe and are affected in epileptic syndromes (Gharaylou et al., 2019). The forceps minor is located at the knee of the corpus callosum and is responsible for connecting the anterior frontal lobes of both hemispheres. Partial epileptic activity in the cerebral hemisphere can cause global epilepsy because it can spread to the contralateral hemisphere through the corpus callosum (Du et al., 2014). The lesions of the aforementioned fiber tracts involve commissural fibers and projection fibers, providing a pathophysiological basis for the accompanying affective disorders, impaired cognitive functions, and motor-related symptoms in epileptic patients.

Diffusion tensor imaging has been widely used in the study of central nervous systems diseases such as acute ischemic cerebral infarction, brain tumors, and multiple sclerosis (Lampinen et al., 2020). The advantage of DTI in studying epilepsy is that it can effectively detect epilepsy lesions that cannot be detected by conventional MRI, reveal the underlying pathophysiology of epilepsy, and provide more information to aid the diagnosis and surgical treatment of epilepsy.

There are also some limitations of this study. First, the sample size was relatively small. Therefore, the results must be regarded as preliminary results. In addition, due to the small sample size of this study, no further intragroup comparison was conducted among epileptic patients in this study. Some studies have shown that there is no significant difference in white matter microstructural damage among different epilepsy subgroups (Focke et al., 2014). Larger sample sizes are needed for verification by future studies, including those aiming to characterize the features of DTI connections in different epilepsy subtypes (Stasenko et al., 2022). Although we confirmed changes in brain network function in epilepsy patients at the cross-sectional level, longitudinal follow-up is still required to reveal changes in brain network function with progression of the disease. Second, although using the same scanner with the same parameters and same operators helps improve the objectivity of our findings, additional sequences in the opposite reading direction are still necessary. We will continue to improve the quality of our research in the next stage, including the use of TOPUP correction through additional reverse sequence scans.

In conclusion, the results of this study suggest that whole-brain structural network analysis by DTI imaging is a feasible method that can be further used to investigate abnormal structural connectivity associated with epilepsy comorbidities. However, as different research methods are irreplaceable, the combination of DTI, fMRI, EEG, and animal experiments may be an ideal research model for clarifying the characteristics and pathogenesis of epilepsy. Inhibiting or disrupting epileptogenic networks could also be a novel approach for treating epilepsy in the future.

## Data availability statement

The original contributions presented in this study are included in the article/supplementary material, further inquiries can be directed to the corresponding authors.

## Ethics statement

The studies involving human participants were reviewed and approved by the Ethics Committee of Suzhou Hospital Affiliated to Nanjing Medical University. The patients/participants provided their written informed consent to participate in this study.

## Author contributions

MW and XC: conception and design of study, analysis and interpretation of data, and drafting the manuscript. QS and BX: conception and design of study and analysis and interpretation of data. XH, HZ, QG, GW, XD, QX, and MS: acquisition of data and analysis and interpretation of data. QC and SX: conception and design of study and drafting the manuscript. HF: conception and design of study, drafting the manuscript, and revising the manuscript. ZD: conception and design of study and revising the manuscript. All authors contributed to the article and approved the submitted version.

## Footnotes

1
http://www.fmrib.ox.ac.uk/fsl

2
http://www.nitrc.org/projects/gretna/

## References

[B1] A Yassine I. M Eldeeb W. A Gad K. A Ashour Y. A Yassine I. O Hosny A. (2018). Cognitive functions, electroencephalographic and diffusion tensor imaging changes in children with active idiopathic epilepsy. *Epilepsy Behav.* 84 135–141. 10.1016/j.yebeh.2018.04.024 29800799

[B2] Bernhardt B. C. Bonilha L. Gross D. W. (2015). Network analysis for a network disorder: The emerging role of graph theory in the study of epilepsy. *Epilepsy Behav.* 50 162–170. 10.1016/j.yebeh.2015.06.005 26159729

[B3] Bullmore E. Sporns O. (2009). Complex brain networks: Graph theoretical analysis of structural and functional systems. *Nat. Rev. Neurosci.* 10 186–198. 10.1038/nrn2575 19190637

[B4] Busceti C. L. Biagioni F. Aronica E. Riozzi B. Storto M. Battaglia G. (2007). Induction of the Wnt inhibitor, Dickkopf-1, is associated with neurodegeneration related to temporal lobe epilepsy. *Epilepsia* 48 694–705. 10.1111/j.1528-1167.2007.01055.x 17437412

[B5] Chen M. Guo D. Li M. Ma T. Wu S. Ma J. (2015). Critical roles of the direct GABAergic pallido-cortical pathway in controlling absence seizures. *PLoS Comput. Biol.* 11:e1004539. 10.1371/journal.pcbi.1004539 26496656 PMC4619822

[B6] Cui Z. Zhong S. Xu P. He Y. Gong G. (2013). PANDA: A pipeline toolbox for analyzing brain diffusion images. *Front. Hum. Neurosci.* 7:42. 10.3389/fnhum.2013.00042 23439846 PMC3578208

[B7] Du H. Xie B. Lu P. Feng H. Wang J. Yuan S. (2014). Impaired white-matter integrity in photosensitive epilepsy: A DTI study using tract-based spatial statistics. *J. Neuroradiol.* 41 131–135. 10.1016/j.neurad.2013.06.002 24524870

[B8] Fahoum F. Lopes R. Pittau F. Dubeau F. Gotman J. (2012). Widespread epileptic networks in focal epilepsies: EEG-fMRI study. *Epilepsia* 53 1618–1627. 10.1111/j.1528-1167.2012.03533.x 22691174 PMC4492710

[B9] Fang S. Li L. Weng S. Guo Y. Zhang Z. Wang L. (2022). Decreasing shortest path length of the sensorimotor network induces frontal glioma-related epilepsy. *Front. Oncol.* 12:840871. 10.3389/fonc.2022.840871 35252008 PMC8888886

[B10] Focke N. K. Diederich C. Helms G. Nitsche M. A. Lerche H. Paulus W. (2014). Idiopathic-generalized epilepsy shows profound white matter diffusion-tensor imaging alterations. *Hum. Brain Mapp.* 35 3332–3342. 10.1002/hbm.22405 25050427 PMC6869818

[B11] Fransson P. Thompson W. H. (2020). Temporal flow of hubs and connectivity in the human brain. *Neuroimage* 223:117348. 10.1016/j.neuroimage.2020.117348 32898675

[B12] Gharaylou Z. Shafaghi L. Oghabian M. A. Yoonessi A. Tafakhori A. Shahsavand Ananloo E. (2019). Longitudinal effects of bumetanide on neuro-cognitive functioning in drug-resistant epilepsy. *Front. Neurol.* 10:483. 10.3389/fneur.2019.00483 31133976 PMC6517515

[B13] Gong J. Chang X. Jiang S. Klugah-Brown B. Tan S. Yao D. (2017). Microstructural alterations of white matter in juvenile myoclonic epilepsy. *Epilepsy Res.* 135 1–8. 10.1016/j.eplepsyres.2017.04.002 28549335

[B14] Hatton S. N. Huynh K. H. Bonilha L. Abela E. Alhusaini S. Altmann A. (2020). White matter abnormalities across different epilepsy syndromes in adults: An ENIGMA-Epilepsy study. *Brain* 143 2454–2473. 10.1093/brain/awaa200 32814957 PMC7567169

[B15] Hu C. Y. Gao X. Long L. Long X. Liu C. Chen Y. (2017). Altered DMN functional connectivity and regional homogeneity in partial epilepsy patients: A seventy cases study. *Oncotarget* 8 81475–81484. 10.18632/oncotarget.20575 29113406 PMC5655301

[B16] Hu Y. Xu Q. Shen J. Li K. Zhu H. Zhang Z. (2015). Small-worldness and gender differences of large scale brain metabolic covariance networks in young adults: A FDG PET study of 400 subjects. *Acta Radiol.* 56 204–213. 10.1177/0284185114529106 24763919

[B17] Ji J. L. Spronk M. Kulkarni K. Repovs G. Anticevic A. Cole M. W. (2019). Mapping the human brain’s cortical-subcortical functional network organization. *Neuroimage* 185 35–57. 10.1016/j.neuroimage.2018.10.006 30291974 PMC6289683

[B18] Jiang W. Li J. Chen X. Ye W. Zheng J. (2017). Disrupted structural and functional networks and their correlation with alertness in right temporal lobe epilepsy: A graph theory study. *Front. Neurol.* 8:179. 10.3389/fneur.2017.00179 28515708 PMC5413548

[B19] Konomi T. Fujiyoshi K. Hikishima K. Komaki Y. Tsuji O. Okano H. J. (2012). Conditions for quantitative evaluation of injured spinal cord by in vivo diffusion tensor imaging and tractography: Preclinical longitudinal study in common marmosets. *Neuroimage* 63 1841–1853. 10.1016/j.neuroimage.2012.08.040 22922169

[B20] Lampinen B. Zampeli A. Bjorkman-Burtscher I. M. Szczepankiewicz F. Kallen K. Compagno Strandberg M. (2020). Tensor-valued diffusion MRI differentiates cortex and white matter in malformations of cortical development associated with epilepsy. *Epilepsia* 61 1701–1713. 10.1111/epi.16605 32667688 PMC7963222

[B21] Liu M. Chen Z. Beaulieu C. Gross D. W. (2014). Disrupted anatomic white matter network in left mesial temporal lobe epilepsy. *Epilepsia* 55 674–682. 10.1111/epi.12581 24650167

[B22] McGill M. L. Devinsky O. Kelly C. Milham M. Castellanos F. X. Quinn B. T. (2012). Default mode network abnormalities in idiopathic generalized epilepsy. *Epilepsy Behav.* 23 353–359. 10.1016/j.yebeh.2012.01.013 22381387 PMC4407647

[B23] Mears D. Pollard H. B. (2016). Network science and the human brain: Using graph theory to understand the brain and one of its hubs, the amygdala, in health and disease. *J. Neurosci. Res.* 94 590–605. 10.1002/jnr.23705 26771046

[B24] Menassa D. A. Sloan C. Chance S. A. (2017). Primary olfactory cortex in autism and epilepsy: Increased glial cells in autism. *Brain Pathol.* 27 437–448. 10.1111/bpa.12415 27409070 PMC8029489

[B25] Nemzer L. R. Cravens G. D. Worth R. M. Motta F. Placzek A. Castro V. (2020). Critical and Ictal phases in simulated EEG signals on a small-world network. *Front. Comput. Neurosci.* 14:583350. 10.3389/fncom.2020.583350 33488373 PMC7820784

[B26] Park K. M. Lee B. I. Shin K. J. Ha S. Y. Park J. Kim S. E. (2019). Pivotal role of subcortical structures as a network hub in focal epilepsy: Evidence from graph theoretical analysis based on diffusion-tensor imaging. *J. Clin. Neurol.* 15 68–76. 10.3988/jcn.2019.15.1.68 30618219 PMC6325361

[B27] Posner M. I. Sheese B. E. Odludas Y. Tang Y. (2006). Analyzing and shaping human attentional networks. *Neural Netw.* 19 1422–1429. 10.1016/j.neunet.2006.08.004 17059879

[B28] Royer J. Bernhardt B. C. Lariviere S. Gleichgerrcht E. Vorderwulbecke B. J. Vulliemoz S. (2022). Epilepsy and brain network hubs. *Epilepsia* 63 537–550. 10.1111/epi.17171 35092011

[B29] Sarnat H. B. Flores-Sarnat L. (2016). Might the olfactory bulb be an origin of olfactory auras in focal epilepsy?. *Epileptic Disord.* 18 344–355. 10.1684/epd.2016.0869 27818364

[B30] Shah A. Lenka A. Saini J. Wagle S. Naduthota R. M. Yadav R. (2017). Altered brain wiring in Parkinson’s disease: A structural connectome-based analysis. *Brain Connect.* 7 347–356. 10.1089/brain.2017.0506 28595456

[B31] Shigemoto Y. Sato N. Sone D. Maikusa N. Yamao T. Kimura Y. (2021). Single-subject gray matter networks in temporal lobe epilepsy patients with hippocampal sclerosis. *Epilepsy Res.* 177:106766. 10.1016/j.eplepsyres.2021.106766 34534926

[B32] Shu N. Liu Y. Li K. Duan Y. Wang J. Yu C. (2011). Diffusion tensor tractography reveals disrupted topological efficiency in white matter structural networks in multiple sclerosis. *Cereb. Cortex* 21 2565–2577. 10.1093/cercor/bhr039 21467209

[B33] Sporns O. (2014). Contributions and challenges for network models in cognitive neuroscience. *Nat. Neurosci.* 17 652–660. 10.1038/nn.3690 24686784

[B34] Stasenko A. Lin C. Bonilha L. Bernhardt B. C. McDonald C. R. (2022). Neurobehavioral and clinical comorbidities in epilepsy: The role of white matter network disruption. *Neuroscientist* [Epub ahead of print]. 10.1177/10738584221076133 35193421 PMC9393207

[B35] Svob C. Wang Z. Weissman M. M. Wickramaratne P. Posner J. (2016). Religious and spiritual importance moderate relation between default mode network connectivity and familial risk for depression. *Neurosci. Lett.* 634 94–97. 10.1016/j.neulet.2016.10.009 27717831 PMC5097884

[B36] Tong X. An D. Xiao F. Lei D. Niu R. Li W. (2019). Real-time effects of interictal spikes on hippocampus and amygdala functional connectivity in unilateral temporal lobe epilepsy: An EEG-fMRI study. *Epilepsia* 60 246–254. 10.1111/epi.14646 30653664

[B37] Tzourio-Mazoyer N. Landeau B. Papathanassiou D. Crivello F. Etard O. Delcroix N. (2002). Automated anatomical labeling of activations in SPM using a macroscopic anatomical parcellation of the MNI MRI single-subject brain. *Neuroimage* 15 273–289. 10.1006/nimg.2001.0978 11771995

[B38] Wu K. Taki Y. Sato K. Qi H. Kawashima R. Fukuda H. (2013). A longitudinal study of structural brain network changes with normal aging. *Front. Hum. Neurosci.* 7:113. 10.3389/fnhum.2013.00113 23565087 PMC3615182

[B39] Xu S. W. Xi J. H. Lin C. Wang X. Y. Fu L. Y. Kralik S. F. (2018). Cognitive decline and white matter changes in mesial temporal lobe epilepsy. *Medicine.* 97:e11803. 10.1097/MD.0000000000011803 30113469 PMC6113048

[B40] Xu S. Yao X. Han L. Lv Y. Bu X. Huang G. (2021). Brain network analyses of diffusion tensor imaging for brain aging. *Math. Biosci. Eng.* 18 6066–6078. 10.3934/mbe.2021303 34517523

[B41] Yao L. Y. Guo Y. C. Zhan X. J. Sun Z. F. Li Y. Wei Y. X. (2018). [Preliminary study of DTI on cerebral white matter micro-structure of patients with idiopathic olfactory loss]. *Lin Chung Er Bi Yan Hou Tou Jing Wai Ke Za Zhi* 32 435–438. 10.13201/j.issn.1001-1781.2018.06.009 29737739

[B42] Young J. C. Vaughan D. N. Nasser H. M. Jackson G. D. (2019). Anatomical imaging of the piriform cortex in epilepsy. *Exp. Neurol.* 320:113013. 10.1016/j.expneurol.2019.113013 31323251

[B43] Zalesky A. Fornito A. Bullmore E. T. (2010). Network-based statistic: Identifying differences in brain networks. *Neuroimage* 53 1197–1207. 10.1016/j.neuroimage.2010.06.041 20600983

[B44] Zhang X. Zhang G. Ma K. Yu T. Xu C. Yan X. (2019). A case of right orbitofrontal epilepsy featuring ictal swearing. *J. Neurol Sci.* 397 1–3. 10.1016/j.jns.2018.12.004 30544099

[B45] Zhu J. Xu C. Zhang X. Qiao L. Wang X. Zhang X. (2021). The changes in the topological properties of brain structural network based on diffusion tensor imaging in pediatric epilepsy patients with vagus nerve stimulators: A graph theoretical analysis. *Brain Dev.* 43 97–105. 10.1016/j.braindev.2020.07.006 32713660

